# Prevalence of multiple roots and complex canal morphology in mandibular premolars among a selected Southern Egyptian sub-population: a CBCT-analysis

**DOI:** 10.1007/s10266-024-00903-7

**Published:** 2024-02-13

**Authors:** Mohamed Ahmed Elsayed, Maii Youssef Elmesellawy, Edgar Schäfer

**Affiliations:** 1 Department of Endodontics, RAK College of Dental Sciences, RAK Medical and Health Sciences University, Ras Al-Khaimah, United Arab Emirates; 2 Department of Endodontics, Faculty of Dentistry, Assiut University, Assiut, Egypt; 3 Endodontic Department, Faculty of Dentistry, Beni-Seuf University, Beni Suef, Egypt; 4 Central Interdisciplinary Ambulance, School of Dentistry, University of Münster, Waldeyerstr. 30, 48149 Münster, Germany

**Keywords:** Cone beam computed tomography, Mandibular premolars, Root canal configuration, Southern Egyptian subpopulation, Vertucci’s classification

## Abstract

The mandibular premolars can pose a significant challenge in root canal treatment due to their complex canal system. This study investigated the prevalence of multiple roots and complex canal morphology of mandibular premolars in a selected Egyptian sub-population using cone beam computed tomography (CBCT). 283 CBCT scans (131 males, 152 females, age 18–70) included 1132 mandibular premolars (566 first, and 566 second premolars) were viewed for incidences ofvariation in root numbers and canal configuration according to Vertucci’s classification. CBCT images were assessed by two endodontists, data were statistically analyzed using Fisher exact and Chi-square tests. The majority of first premolars (85.7%) exhibited a single root, whereas 14.7% had 2 roots with a significantly higher frequency in males (19.8%) than in females (9.5%) (*P* < .05). The most prevalent type was type I (57.8%), followed by type V (21.7%), while types II and VII made up only 1%. Types V and III were more prevalent among females, while males had a higher prevalence of types I and IV. In 2.5% of cases, mandibular second premolars were found to have 2 roots, with a higher incidence in males (*P* < .05). Type I canals were significantly more prevalent (90.8%) than other types, followed by type V (5.3%) (*P* < .05). A statistically significant gender correlation was found regarding root number and canal configuration. It is not uncommon to find mandibular first premolars with two roots in the southern Egyptian population, particularly in males. These observations may be valuable for dentists who treat Southern Egyptians, in Egypt and other countries.

## Introduction

In endodontic practice, it is essential for clinicians to have an understanding of root canal anatomical variations, as fully acquainting common root canal morphology enables the clinician to perform cleaning and shaping procedures more efficiently with fewer procedural errors [1]. Additionally, using cone beam computed tomography (CBCT) for the pre-evaluation of root canal morphology provides a better understanding of the challenges that the clinician might encounter, which can ultimately lead to improved treatment outcomes and prognosis [2].

Owing to the disparity of root canal morphology among diverse populations, several studies [3, 4] have been conducted using various techniques to recognize and correlate these variations with different populations and ethnic groups. Techniques such as dye penetration, sectioning, electron microscopy, canal staining, tooth clearing [5], intra-radicular contrast medium radiography [6], and micro-computed tomography (micro-CT), which has been referred to as the gold standard [7, 8], have been utilized in previous studies. These techniques are ex vivo and are only applicable to extracted teeth [9]. While other techniques such as conventional 2D radiography, operating microscope, and CBCT are reliable for clinical use [10].

Earlier studies [11–13], recognized high rates of morphological variations in mandibular premolars, this diversity of canal anatomy is genetically and ethnically based in the first place [14]. Few studies have examined the Egyptian population [15, 16], however, this is the first study to focus solely on the teeth morphology of a southern Egyptian subpopulation. Considering the anatomical variations during root canal treatment of southern Egyptians is of great significance and could improve treatment outcomes.

## Materials and methods

The protocol of this retrospective study was approved by the Ethics Review Committee at Beni-Suef University, Egypt (# REC-FDBSU/03112022-01/EM). A power analysis was designed to have adequate power to apply statistical analysis regarding the prevalence of multiple roots of mandibular premolars in the southern Egyptian sub-population using CBCT. By adopting a confidence interval of 95%, a margin of error of 5% with finite population correction and by using a prevalence of multiple roots of mandibular premolars of 16%, based on the results of a pilot study, the predicted sample size (n) was 206 cases. Sample size calculation was performed using Epi info for Windows version 7.2 (CDC, Atlanta, GA, USA). A total of 561 CBCT scans that had been taken for diagnostic purposes between 2019 and 2021 were retrospectively collected from radiology centers in the city of Assiut in southern Egypt.

### Inclusion and exclusion criteria

The inclusion criteria specified Egyptian patients aged between 18 and 70 years, whereas exclusion criteria involved scans with a missing mandibular premolar, internal resorption, root canal fillings, and post and crown restorations. A total of 283 scans (131 males, 152 females) were included in the study after the application of these criteria, encompassing 1132 mandibular premolars, evenly distributed between 566 mandibular first premolars and 566 mandibular second premolars.

### Image acquisition and analysis

All scans were taken either by(Papaya X; GENORAY, Seoul, Korea) with a field of view (FOV) of 14 × 14 cm and voxel size ranging between 80 and 200 µm, or by (NewTom VGi evo; NewTom, Verona, Italy) with FOV of 24 × 19 cm, and voxel size ranged between 150 and 300 µm. Analysis of the CBCT scans was done distinctly twice by two endodontists with almost 10 years of experience using CBCT. In the beginning, both inter and intra-examiner reliability were determined by analyzing 20 scans, the value of Cohen’s kappa for the inter-observer agreement was 0.86. A code has been given for all scans, and any disagreement between the examiners (19 out of 283 cases) was discussed until a consensus was reached. To avoid any bias, all scans were consecutively numbered and that was the only information available for both examiners.

**Fig. 1:**
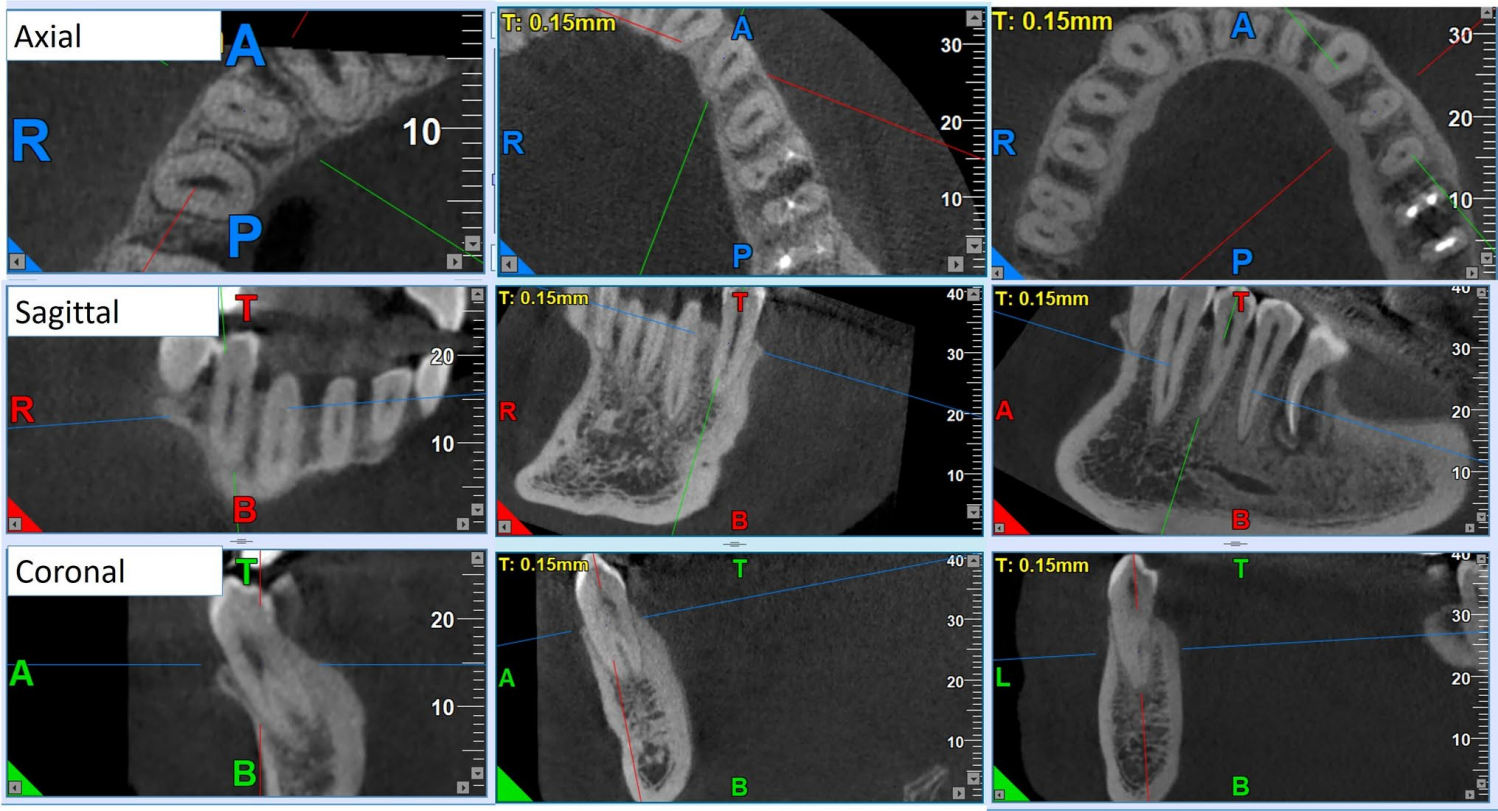
CBCT images of multirooted mandibular first premolars in axial, sagittal, and coronal views

**Fig. 2:**
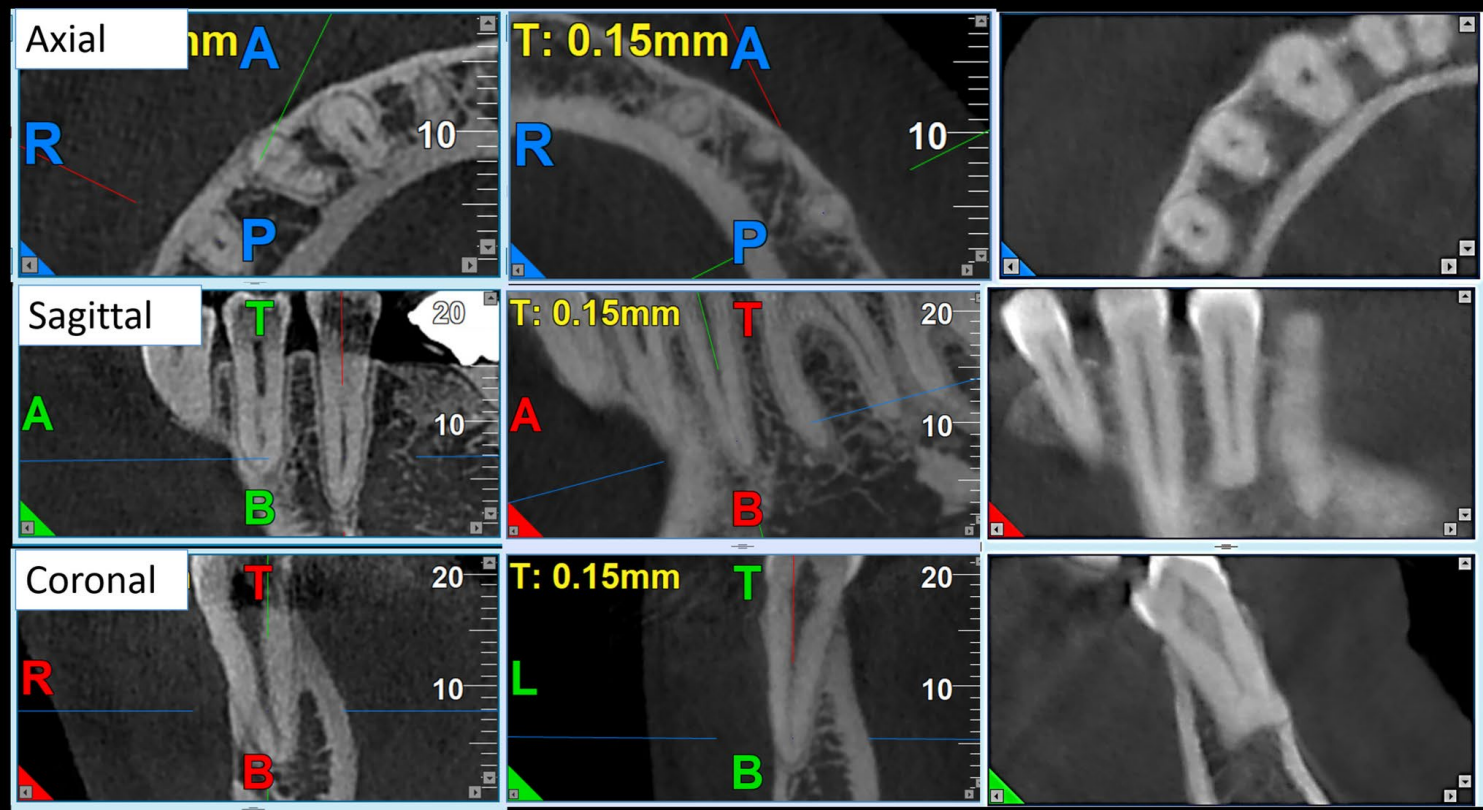
CBCT images of single-rooted mandibular second premolars in axial, sagittal, and coronal views

The CBCT images were viewed in three planes (axial, sagittal, and coronal) to assess the number of roots, root canal configuration, and bilateral symmetry (Figs. 1, 2). To assess the root numbers and canal configuration it was necessary to examine any tooth in different orientations and angles. However, the primary focus was on the axial view, which required a slow and gradual scrolling of the toolbar from the pulp chamber to the apex. Vertucci's classification was utilized to assess the root canal configuration. This classification system is designed to describe and classify the various morphological patterns of the root canal system into eight types to encompass complex configurations [5].

### Statistical analysis

The frequency and percentage of additional roots and different types of canals in relation to gender were assessed, and the data were analyzed using the chi-square test. Pairwise comparisons were conducted using multiple Fisher’s exact tests with p-value adjustment through the false discovery rate (FDR) method. The statistical analysis was performed using R statistical analysis software version 4.1.3 for Windows (R Core, Vienna, Austria-https://www.R-project.org). *P* values < 0.05 were considered significant.

## Results

A total of 1132 mandibular premolars were assessed in this study. One-rooted mandibular first premolars occurred significantly more often in both genders (85.4%) than two-rooted ones (14.7%) (*P* < 0.05). In males, however, the most common canal configuration was type I, followed by type V and IV; on the other hand, females showed the highest incidence of type I canals followed by types V and III. Both genders rarely exhibited type II and type VII. The incidence of one root was significantly higher in females (90.5%) than in males (80.2%) (X^2^ = 12.19, *P* < 0.05). Conversely, the incidence of two roots was significantly lower (14.6%) with a greater frequency among males (19.8%) than in females (9.5%) (Table 1) (*P* < 0.05). The mandibular second premolar demonstrated a significantly higher incidence of single-rooted teeth (96.5%) than 2-roots (2.5%), while three rooted premolars were very rare (1%) (*P* < 0.05). A higher occurrence of single-rooted teeth was observed in females (98.7%) than in males (93.9%) (X^2^ = 10.09, *P* < 0.05). On the contrary, the frequency of two-rooted premolars was higher in males (4.6%) than in females (0.7%) (*P* < 0.05). Canal types I and V occurred more frequently than other canal configurations in both genders (Table 2). Mandibular first and second premolars showed bilateral symmetry regarding the number of roots and canal configurations (Table 3).

**Table 1:** The distribution and percentage of the number of roots and canal configurations in the mandibular first premolar

			Number of roots					Canal configuration									
		Total number of teeth	Single root	2 roots	3 roots	χ^2^	p-value	Type (I)	Type (II)	Type (III)	Type (IV)	Type (V)	Type (VI)	Type (VII)	Type (VIII)	χ^2^	p-value
Male	n (%)	262 (100%)	210^A^ (80.2%)	52^B^ (19.8%)	0 (0%)	12.19	< 0.001*	159^A^ (60.7%)	2^D^ (0.8%)	16^C^ (6.1%)	36^B^ (13.7%)	49^B^ (18.7%)	0 (0.0%)	0 (0.0%)	0 (0.0%)	29.46	< 0.001*
Female	n (%)	304 (100%)	275^A^ (90.5%)	29^B^ (9.5%)	0 (0.0%)			168^A^ (55.3%)	4^E^ (1.3%)	39^C^ (12.8%)	13^D^ (4.3%)	74^B^ (24.3%)	0 (0.0%)	6 (2.0%)	0 (0.0%)		
Total	n (%)	566 (100%)	485^A^ (85.4%)	81^B^ (14.7%)	0 (0%)			327^A^ (57.8%)	6^D^ (1.0%)	55^C^ (9.5%)	49^C^ (9.0%)	123^B^ (21.7%)	0 (0%)	6^D^ (1.0%)	0 (0%)		

Different superscript letters indicate a statistically significant difference within the same heading row (*P* < 0.05)

**Table 2:** The distribution and percentage of the number of roots and canal configurations in the mandibular second premolar

			Number of roots					Canal configuration									
		Total number of teeth	Single root	2 roots	3 roots	χ^2^	p-value	Type (I)	Type (II)	Type (III)	Type (IV)	Type (V)	Type (VI)	Type (VII)	Type (VIII)	χ^2^	p-value
Male	n (%)	262 (100%)	246^A^ (93.9%)	12^B^ (4.6%)	4^B^ (1.5%)	10.09	0.006*	242^A^ (92.4%)	0 (0.0%)	0 (0.0%)	2^C^ (0.8%)	14^B^ (5.3%)	2^C^ (0.8%)	0 (0.0%)	2^C^ (0.8%)	12.84	0.046*
Female	n (%)	304 (100%)	300^A^ (98.7%)	2^B^ (0.7%)	2^B^ (0.7%)			272^A^ (89.5%)	4^C^ (1.3%)	8^BC^ (2.6%)	2^C^ (0.7%)	16^B^ (5.3%)	0 (0.0%)	0 (0.0%)	2^C^ (0.7%)		
Total	n (%)	566 (100%)	546^A^ (96.5%)	14^B^ (2.5%)	6^B^ (1.0%)			514^A^ (90.8%)	4^C^ (0.7%)	8^C^ (1.4%)	4^C^ (0.7%)	30^B^ (5.3%)	2^C^ (0.4%)	0 (0.0%)	4^C^ (0.7%)		

Different superscript letters indicate a statistically significant difference within the same heading row (*P* < 0.05)

**Table 3:** Distribution and Percentage of Roots and Canal Configurations in Mandibular First and Second Premolars on Right and Left Sides

Tooth	Side		Total Number of teeth	Number of roots			Canal configuration							
				Single root	2 roots	3 roots	Type (I)	Type (II)	Type (III)	Type (IV)	Type (V)	Type (VI)	Type (VII)	Type (VIII)
First premolar	Right	n (%)	283 (100%)	243 (85.9%)	40 (14.1%)	0 (0.0%)	164 (58.0%)	4 (1.4%)	28 (9.9%)	28 (9.9%)	57 (20.1%)	0 (0.0%)	2 (0.7%)	0 (0.0%)
	Left	n (%)	283 (100%)	242 (85.5%)	41 (14.5%)	0 (0.0%)	163 (57.6%)	2 (0.7%)	27 (9.5%)	21 (7.4%)	66 (23.3%)	0 (0.0%)	4 (1.4%)	0 (0.0%)
Second premolar	Right	n (%)	283 (100%)	273 (96.5%)	6 (2.1%)	4 (1.4%)	253 (89.4%)	2 (0.7%)	4 (1.4%)	4 (1.4%)	14 (4.9%)	2 (0.7%)	0 (0.0%)	4 (1.4%)
	Left	n (%)	283 (100%)	273 (96.5%)	8 (2.8%)	2 (0.7%)	261 (92.2%)	2 (0.7%)	4 (1.4%)	0 (0.0%)	16 (5.7%)	0 (0.0%)	0 (0.0%)	0 (0.0%)

## Discussion

Having a comprehensive understanding of the internal morphology of mandibular premolars and their anatomic variations is crucial for endodontic treatment. Earlier studies [17, 18] reported extremely variable and complex internal anatomy in mandibular premolars, with a high chance of extra canals. Racial and ethnic variations play a major role in the morphological complexities of teeth and previous studies observed substantial differences in the number of roots and internal morphology of premolars between different populations and ethnicities as in the Middle Eastern, Caucasian, Indian, and Chinese [5, 19, 20]. Complex root canal anatomy cannot be accurately detected through two-dimensional radiographic images, hence, it is essential to rely on more advanced tools such as CBCT, which provides three-dimensional images with a higher level of resolution. CBCT gives a more comprehensive understanding of the root canal anatomy while minimizing radiation exposure [21], and is a non-invasive alternative to traditional methods such as clearing and dye penetration techniques [22].

Using 283 CBCT scans, this study investigated the frequency of root numbers and root canal morphology in mandibular premolars among a selected southern Egyptian population according to Vertucci's classifications [5]. A total of 566 mandibular first and 566 mandibular second premolars were examined in the analysis. In the current study, single-rooted mandibular first premolars were found in 85.4% of the cases, which is in agreement with incidences ranging from 85.0 to 99.9% reported in a previous study [18], and values reported for Spanish [23], Turkish [24], and Korean populations [25]. However, the southern Egyptian population exhibited a higher prevalence of two-rooted mandibular first premolars (14.7%) compared to other ethnic groups, such as Saudi (3.1%) [12], German (8.6%) [26], western Chinese (2%) [27], Thai (4.87%) [28], Iranian (14.4%) [29], and Korean populations (0.1%) [25]. It is important to note that different techniques have produced varying results in determining the prevalence of the two-rooted mandibular first premolars. For example, in the Egyptian population [30], decalcifying and clearing techniques showed that only 3.2% of teeth had two roots, while two-dimensional radiography revealed a slightly higher incidence of 16.2% in the African American population [19]. These conflicting results may be attributed to differences in analysis techniques and various subpopulations.

When analyzing the canal configuration of mandibular first premolars, it was found that 57.8% of teeth had a single canal (Vertucci's type I), while type V and type III accounted for 21.7% and 9.5%, respectively. These results are consistent with a prior study conducted on the Egyptian population [30], and a recent meta-analysis [20]. Other CBCT analysis studies have shown that the Chinese population had 54% of first mandibular premolars with a single canal (type I) [31], the Thai population 63% [32], the South Indian population 83.81% [11], and the Turkish population [24] 93.5% mandibular first premolars with Vertucci's type I. A review [18] revealed that 75.8% of teeth had a single canal, while two or more canals were found in 24.2% of teeth. Trope et al. [19] reported that African Americans had a 32.8% occurrence of mandibular first premolars with two or more canals.

In the current study, the vast majority (96.5%) of mandibular second premolars had only one root, while 2.5% had two roots. These results are consistent with previous studies, conducted using CBCT, which found a high prevalence of single-rooted second premolars in Saudi (95.6%) [12], Turkish (98.5%) [24], German (98.16%) [26], and Korean populations (99.9%) [25]. Additionally, the most common canal configuration among mandibular second premolars was type I (90.8%), followed by type V and III with an incidence of 5.3% and 1.4%, respectively, which is corroborated by previous CBCT studies in German [26], Turkish [24], and Spanish populations [23]. Comparable percentages were found in the Jordanian population [33], utilizing the clearing technique. In the present study, types II, IV, and VIII were rarely observed (0.7%).

Gender differences regarding the canal morphology of mandibular premolars have been reported in several studies [12, 34, 35]. In the present study, an association was observed between gender and the number of roots, as a higher frequency of single-rooted teeth was particularly observed in females, which is consistent with a recent review [20]. Moreover, two-rooted mandibular premolars were more prevalent in the male than in the female population, this was similarly observed in a Portuguese subpopulation [36], as females had a lower number of roots per tooth in all teeth compared to males. However, in the present study, the occurrence of three roots was a rare finding in both genders, with marginal differences between males and females in the mandibular second premolars. On the other hand, the occurrence of two canals in the first premolars was more prevalent in females. In the current study, a bilateral symmetry was observed for mandibular first and second premolars in terms of the number of roots and canal configurations, and this is in agreement with a previous study in Norway [37]. On the contrary, another study in the Turkish population showed that the incidence of type I configuration was higher on the right compared to the left side [24].

This study has some limitations, including the small sample size and the use of CBCT records collected by different machines with varying image resolution and voxel sizes (ranging from 150 to 300 µm). Moreover, a selected population was used for this study. Diagnostic purposes were the indications for CBCT analysis. However, a cross-sectional distribution can be assumed as there was no focus on any endodontic treatment planning. These factors should be considered when interpreting the study results. Furthermore, there are limited published data on the teeth morphology of certain populations and geographic groups in Africa. Therefore, more studies are needed in this area using non-invasive methods to address these gaps in knowledge.

## Conclusions

The findings of this study point out that in the southern Egyptian sub-population, there are variations in root number and canal morphology of mandibular premolars, predominated by one-rooted teeth. Two-rooted mandibular first premolars showed a significantly higher prevalence in males than in females. Vertucci’s type I succeeded by type V, and type III was most common in mandibular premolars.
