# Correction: Prevalence of multiple roots and complex canal morphology in mandibular premolars among a selected Southern Egyptian sub-population: a CBCT-analysis

**DOI:** 10.1007/s10266-024-00919-z

**Published:** 2024-02-26

**Authors:** Mohamed Ahmed Elsayed, Maii Youssef Elmesellawy, Edgar Schäfer

**Affiliations:** 1 Department of Endodontics, RAK College of Dental Sciences, RAK Medical and Health Sciences University, Ras Al-Khaimah, United Arab Emirates; 2 Department of Endodontics, Faculty of Dentistry, Assiut University, Assiut, Egypt; 3 Endodontic Department, Faculty of Dentistry, Beni-Seuf University, Beni Suef, Egypt; 4 Central Interdisciplinary Ambulance, School of Dentistry, University of Münster, Waldeyerstr. 30, 48149 Münster, Germany

**Correction to: Odontology** 10.1007/s10266-024-00903-7

In the original publication of the article, the title was processed incorrectly during the production process as follows, “Prevalence of mandibular premolars among a selected multiple roots and complex canal morphology in Southern Egyptian sub-population: a CBCT-analysis”.

The correct title should read as follows, “Prevalence of multiple roots and complex canal morphology in mandibular premolars among a selected Southern Egyptian sub-population: a CBCT-analysis”.

The original article was updated.

